# The use of APACHE II, SOFA, SAPS 3, C-reactive protein/albumin ratio, and lactate to predict mortality of surgical critically ill patients: A retrospective cohort study: Erratum

**DOI:** 10.1097/MD.0000000000016675

**Published:** 2019-07-19

In the article, “The use of APACHE II, SOFA, SAPS 3, C-reactive protein/albumin ratio, and lactate to predict mortality of surgical critically ill patients: A retrospective cohort study”,^[1]^ which appearing is Volume 98, Issue 26 of *Medicine*, Mayra Gonçalves Menegueti's name appeared incorrectly as Mayra Gonçalves Menegheti.
